# *Cox7a2l* Variation Influences Complex III and Mitochondrial Supercomplex Differences in 3xTG-AD Mixed-Background Mice

**DOI:** 10.3390/ijms27177710

**Published:** 2026-08-28

**Authors:** Wenzhuo Ma, Clarissa Cavarsan, Lauren F. Bazinet, Scarlett M. Silvia, Joseph Owusu-Sarfo, Janet Atoyan, Judianne Davis, William E. Van Nostrand, John K. Robinson, Richard T. Clements

**Affiliations:** 1 Department of Biomedical and Pharmaceutical Sciences, College of Pharmacy, University of Rhode Island, Kingston, RI 02881, USA; wenzhuo_ma@uri.edu (W.M.); clarissacavarsan@gmail.com (C.C.); lauren.bazinet@uri.edu (L.F.B.); scarhuck@gmail.com (S.M.S.); jowusu-sarfo@uri.edu (J.O.-S.); jatoyan@uri.edu (J.A.); judiannedavis@uri.edu (J.D.); wvannostrand@uri.edu (W.E.V.N.); johnkrobinson@uri.edu (J.K.R.); 2 George and Anne Ryan Institute for Neuroscience, University of Rhode Island, Kingston, RI 02881, USA; 3 Department of Psychology, College of Health Sciences, University of Rhode Island, Kingston, RI 02881, USA; 4 Department of Surgery, Brown University Health, Warren Alpert Medical School of Brown University, Providence, RI 02903, USA; 5 Ocean State Research Institute, Providence VA Medical Center, Providence, RI 02908, USA

**Keywords:** supercomplex, mitochondrial supercomplex, Alzheimer’s disease, respiration, COX7RP, SCAF1

## Abstract

Mitochondrial dysfunction, including impaired respiration and increased reactive oxygen species (ROS), is an early feature of Alzheimer’s disease (AD) models. Electron transport chain supercomplexes (mSCs) regulate respiratory efficiency and ROS generation, yet genetic determinants of mSC organization in AD models remain underappreciated. Cox7a2l is known to promote CIII_2_/CIV association and CIV incorporation into mSCs. We examined the commonly used mixed-background B6;129S 3xTg-AD mice and B6129SF2/J controls aged 12–15 months, as well as specific rat AD models for changes in mSC organization using blue and clear native-PAGE and DIA-MS. BN and CN-PAGE revealed a marked shift toward larger mSC assemblies in 3xTg-AD mice and a distinct ~650 kDa assembly of complex III absent from controls. Gene sequencing showed that 3xTg-AD mice retained the full-length *Cox7a2l* variant similar to 129S strains, whereas controls predominantly carried a shortened C57BL/6-associated variant. DIA-MS identified greatly increased Cox7a2l in differential mSC bands. Similar changes were found in 3xTg-AD heart tissue. In contrast, AD rat models and their controls, all expressing full-length *Cox7a2l*, did not exhibit comparable mSC differences. These findings suggest *Cox7a2l* genotype as a determinant of mSC organization and highlight the need to account for genetic background when interpreting mSC-dependent mitochondrial phenotypes in mixed-background 3xTg-AD studies. This distinction is essential for accurately separating strain-dependent effects from AD-associated mitochondrial changes.

## 1. Introduction

Mitochondrial dysfunction is recognized as an important and early contributor to Alzheimer’s disease (AD) pathology. Mitochondrial alterations are among the earliest detectable changes in cellular and animal models of AD and can precede robust amyloid plaque generation as well as cognitive or behavioral impairments [1,2,3]. Reported perturbations include increased mitochondrial reactive oxygen species (ROS) generation [4], decreased respiration and mitochondrial membrane potential [5,6,7], and structural remodeling associated with imbalanced mitochondrial fission and fusion [8]. These changes are thought to contribute to neuronal vulnerability by impairing bioenergetics, synaptic function, and ultimately cognitive and memory performance [9]. Because many of these endpoints are directly influenced by electron transport chain (ETC) organization, defining factors that regulate mitochondrial respiratory architecture in AD models is essential for interpreting disease-associated mitochondrial phenotypes.

Mitochondrial supercomplexes (mSCs) are higher-order assemblies of physically associated ETC respiratory complexes [10,11]. The minimal functional mSC contains one complex I, a dimer of complex III, and one complex IV (denoted as CI_1_/CIII_2_/CIV_1_), and larger mSC assemblies consist of CI_1_/CIII_2_/CIV_2–3_ and CI_2_/CIII_2_/CIV_2_. Evidence suggests mSCs facilitate more direct electron transfer, thereby reducing ROS generation and improving respiratory efficiency [12,13,14]. The assembly of individual ETC complexes and smaller subassemblies (CI/CIII_2_ or CIII_2_/CIV) into larger mSCs is dynamic and is proposed to rapidly tune mitochondrial energy production to cellular demand [15]. Among known regulators of mSC organization, *Cox7a2l* (COX7RP, SCAF1) is a key supercomplex assembly factor that promotes CIII_2_/CIV_1_ binding, increases CIV mSCs, and modulates respiration and ROS generation in multiple cell types [16,17,18]. *Cox7a2l* expression may be particularly important for mouse studies because inbred strains differ in specific *Cox7a2l* variants: 129S and most other strains carry the full-length functional allele, whereas C57BL/6 carries a variant with a two-amino acid deletion that markedly affects mSC assembly [19]. In contrast, rats, humans, and nearly all other mammals carry the full-length functional *Cox7a2l* sequence.

This strain-dependent biology is directly relevant to the 3xTg-AD mouse model, which is a widely used model incorporating familial AD-associated APP Swedish (KM670/671NL) and PSEN1 M146V mutations together with mutant Tau P301L overexpression, resulting in amyloid deposition and tau pathology that model key histopathological features observed in humans [20,21]. The APP (swe) and MAPT P301L genes are driven by the neuronal mouse Thy1.2 promotor, and PSEN1 mutation is a knock-in. The most used 3xTg-AD line is maintained on a mixed C57BL/6;129S background and is often compared with separately maintained wild-type B6129SF2/J control mice. Here, we tested whether mSC organization is altered in this model and identified a pronounced difference in ETC supramolecular organization between 3xTg-AD mice and their commonly used control strain. Specifically, the 3xTg-AD mice examined retained the 129S *Cox7a2l* variant and exhibited robust formation of a 650 kD complex III assembly and had larger supercomplexes with greater CIV incorporation. Mice from the control B6129SF2/J line more frequently exhibited a supercomplex profile consistent with the shorter C57BL/6-associated *Cox7a2l* variant. B6129SF2/J mice with the longer variant had ETC organization patterns highly similar to the 3xTG-AD mice.

Together, these findings suggest *Cox7a2l* genotype as a major determinant of mSC assembly in a widely used AD mouse model system and highlight an important genetic variable for interpreting mitochondrial phenotypes. Rather than reflecting AD-like pathology alone, differences in mSC formation, respiration, ROS generation, and mitochondrial metabolism in mixed-background 3xTg-AD studies may be strongly influenced by *Cox7a2l*-dependent ETC organization. These results underscore the necessity of *Cox7a2l* variant matching when assessing AD-related changes in mitochondrial function and provide crucial information for the design and interpretation of mitochondrial studies in the 3xTg-AD mice and more broadly other AD mouse models on the C57Bl6 background.

## 2. Results

**Figure 1:**
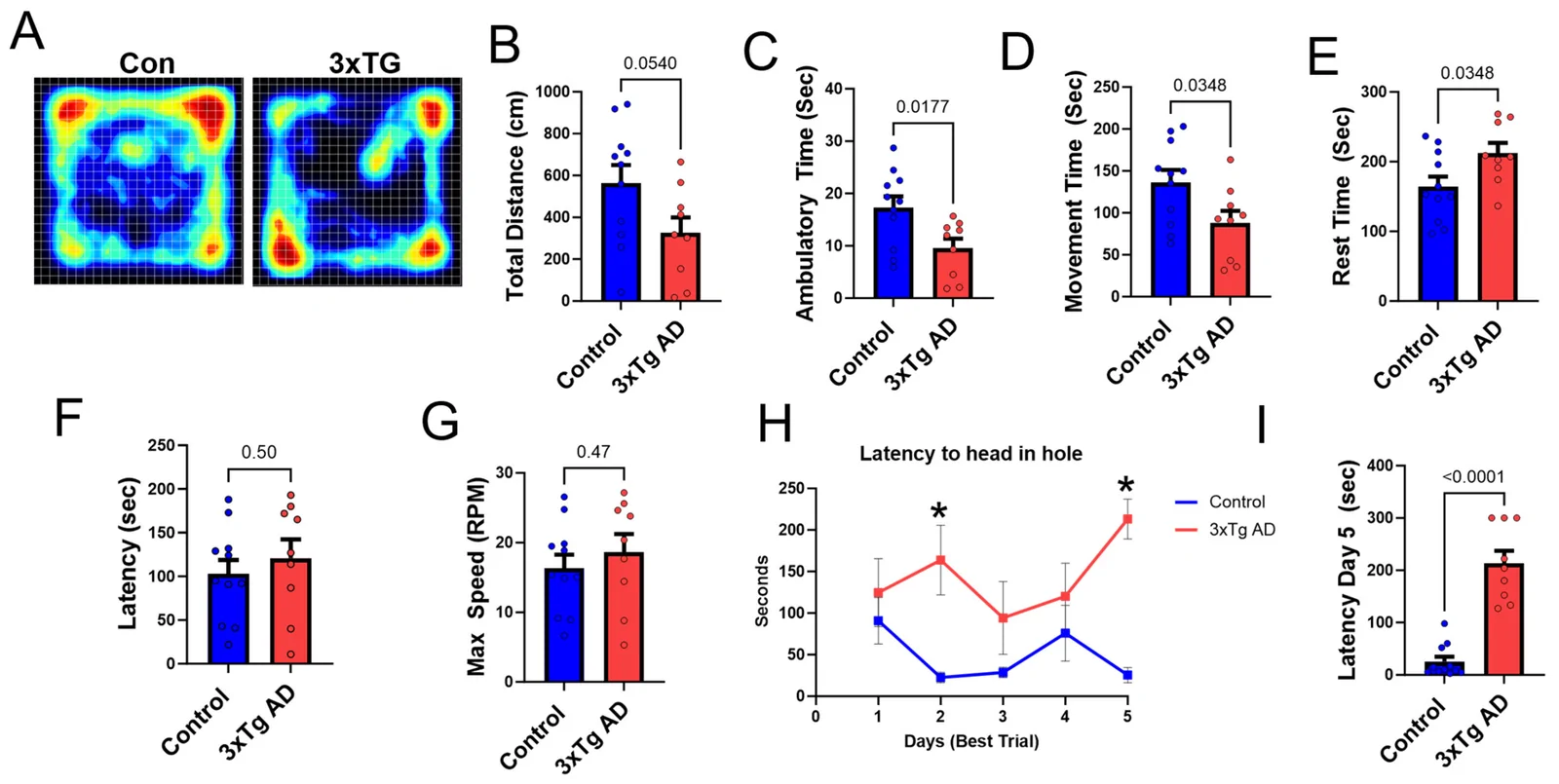
3xTg-AD mice exhibit altered open field behavior and decreased performance in Barnes Maze task. (**A**) Representative movement heatmaps in open field testing for 5 min in control and 3xTg-AD mice. (**B**–**E**) Total distance traveled, ambulatory, movement and rest time in Day 2 of open field maze (control n = 11, 3xTg AD n = 9 *t*-test). (**F**,**G**) Latency to fall and maximum speed in rotarod task in 3xTg-AD and control mice (control n = 11, 3xTg AD n = 9 *t*-test, +/− SEM). (**H**) Time course of latency for first head entry into escape hole in Barnes Maze, (**I**) Quantitation of Day 5 data (control n = 11, 3xTg AD n = 9, * *p* < 0.05 Two Way RM ANOVA, Holm–Sidak post hoc).

Control (B6129SF2/J) and 3xTg-AD mice were tested for behavioral changes consistent with previously reported AD-induced alterations in this model [22,23]. Open Field testing demonstrated reduced exploration, ambulatory and movement time, and decreased distance traveled in the maze (Figure 1A–E). Changes in locomotion were not due to motor deficiencies as 3xTg-AD mice had comparable performance to control mice as assessed by time and maximum RPM achieved in rotorod assay (Figure 1F,G). Memory testing via Barnes maze showed increased latency to finding the escape hole in 3xTg-AD mice vs. control mice (Figure 1H,I), suggesting impaired memory. Main effects of Two-Way RM ANOVA for Barnes Maze data showed *p* < 0.01 for genotype (Con vs. Alz), *p* = 0.28 for Time (Days), and *p* = 0.02 for their interaction.

**Figure 2:**
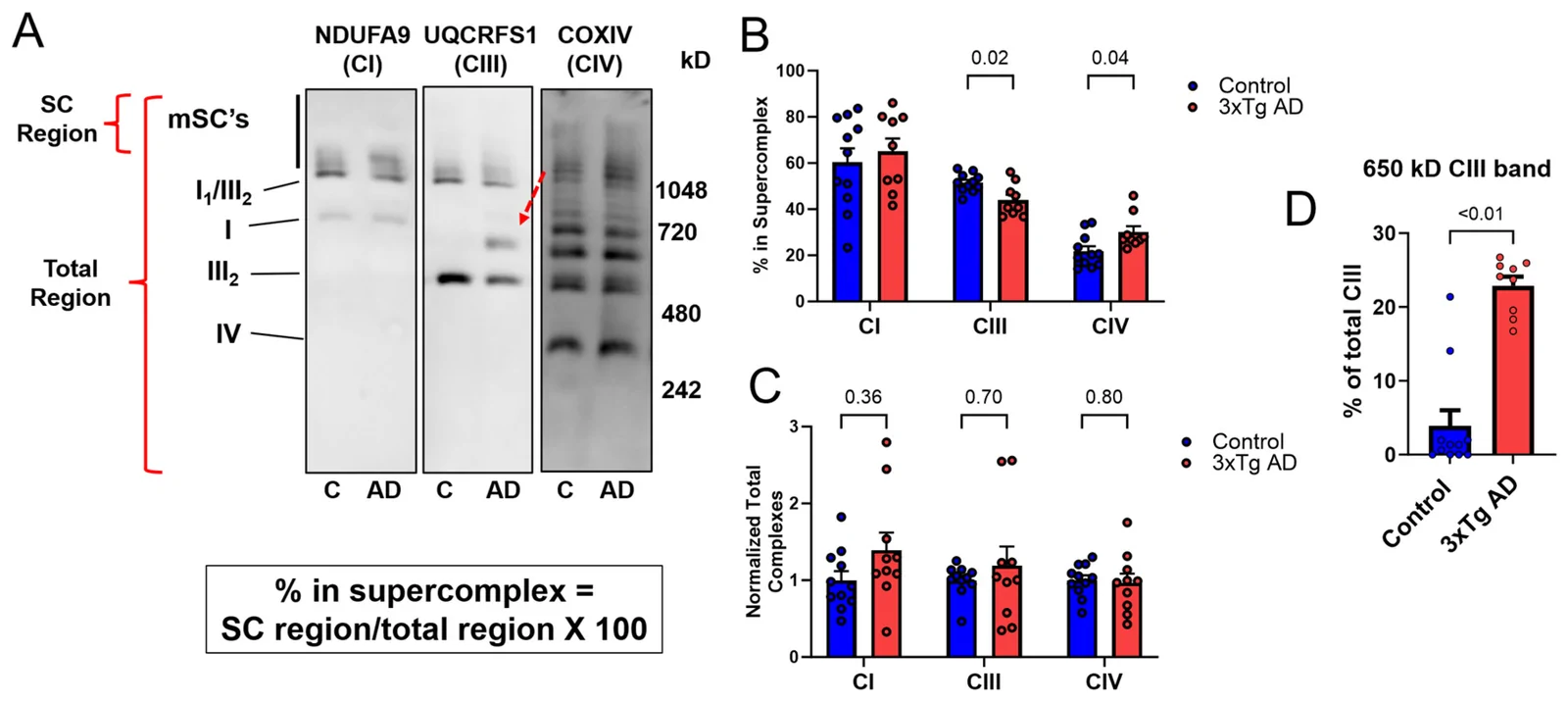
3xTg-AD exhibit altered mSC structure and CIII organization. (**A**) BN-PAGE of control and 3xTg-AD brain mitochondria probed for complex I (NDUFA9), Complex III (UQCRFS1), and Complex IV (COXIV). (**B**) Quantitation of the % of total complex signal in the supercomplex region and (**C**) total complexes normalized to control group. (**D**) Quantitation of the 650 kD CIII band in (**A**) (red arrow) as % of total complex III. *p* values via *t*-test (control n = 11, 3xTg AD n = 9).

Following behavioral testing, mitochondria were isolated from forebrain tissue via differential centrifugation and subjected to blue-native PAGE to assess mitochondrial supercomplexes with immunoblot with antibodies to complex I (NDUFA9), complex III (UQCRFS1), and complex IV (CoxIV) (Figure 2A). Comigration of all three complexes is indicative of mSCs, with the smallest mSC indicative of the I_1_/III_2_/IV_1_ (Figure 2A). Quantitation of the total mSC signal indicated a complicated pattern with small decreases in signal for CIII and increases in CIV in the supercomplex region (labeled I_1_/III_2_/IV_1–n_), with no changes in total assembled complexes (Figure 2B,C). There were also significant changes in the pattern of CIII complexes with a ~650 kD CIII complex present in 3xTg-AD mice but not in control mice, at the apparent expected height of complex III_2_/IV_1_ (Figure 2A dashed arrow and Figure 2D). An additional set of representative gels is provided in Supplemental Figure S1A.

**Figure 3:**
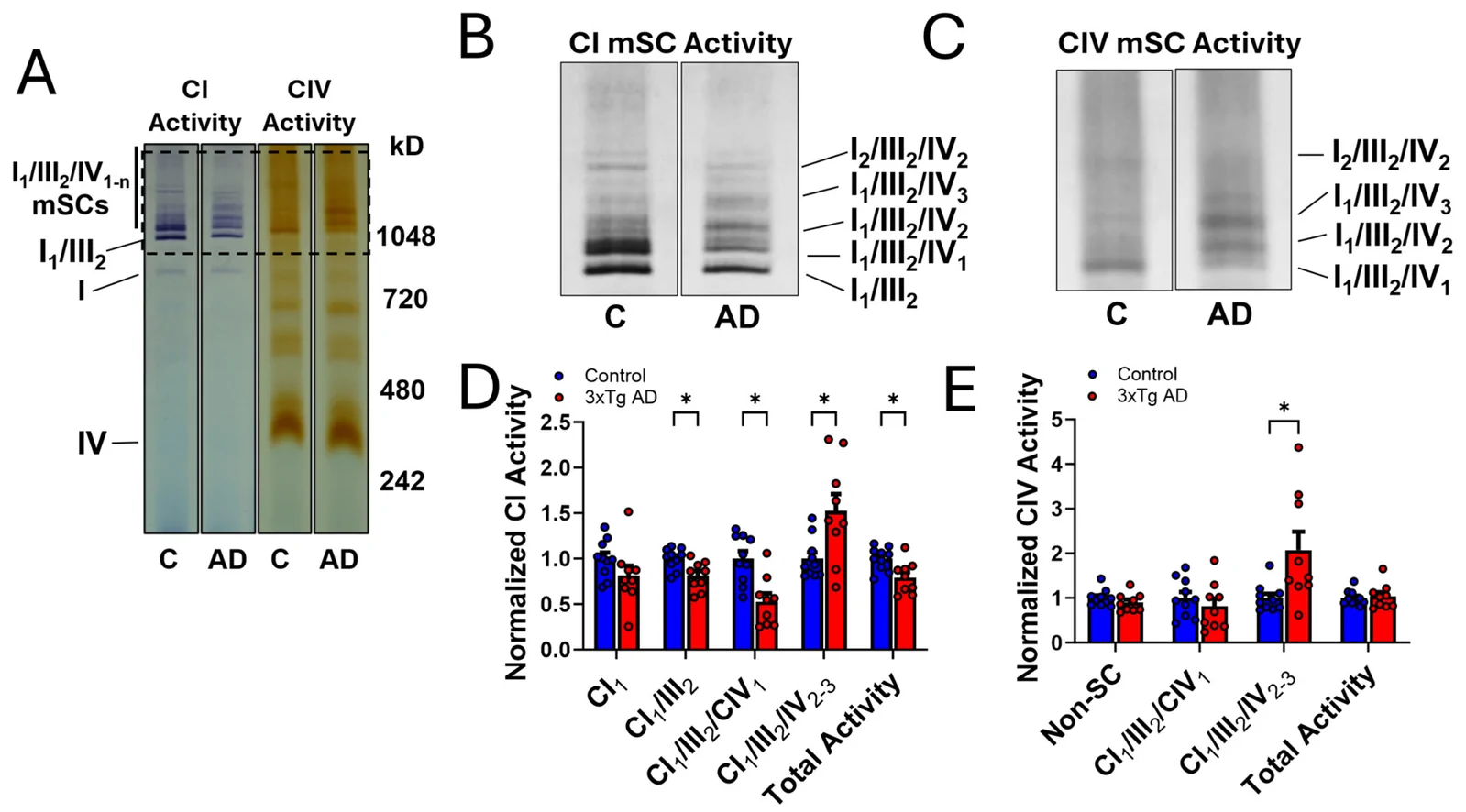
3xTg-AD mice have larger mSC than control mice and less overall complex I activity. (**A**) Representative CN-PAGE of control and 3xTg-AD isolated mitochondria developed for CI in-gel activity (purple) or CIV in-gel activity (brown). (**B**,**C**) Zoomed and contrast-enhanced boxed region in (**A**) showing a decrease in CI_1_/CIII_2_/CIV_1_ mSC containing CI and CIV and an increase in mSC with greater CIV content in 3xTgAD mice (mSC designation based on size and intensity and comigration patterns). (**D**,**E**) Quantitation of bands in (**B**) and (**C**) respectively, normalized to entire protein load assessed by Coomassie stained mitochondrial proteins in gel. (control n = 11, 3xTg AD n = 9, * *p* < 0.05 *t*-test +/− SEM).

To better visualize potential changes in mSC distribution in 3xTg-AD mice, we employed CN-PAGE, which can be used to assess CI and CIV abundance following in-gel colorimetric development of artificial substrates for CI (NTB: purple) and CIV (DAB: brown) and in our experience is more sensitive than antibody-based methods of detection. CN-PAGE provides better resolution and reduced background, allowing quantitation of individual mSC species. Of note, colorimetric in-gel complex I and IV activity assays are not true measures of time-dependent enzyme activity rate but rather semi-quantitative analysis with color development and densitometric signal being partially dependent on potential enzyme activity differences but mostly reflective of differences in complex abundance at a specific location. Representative gels of CI in-gel activity (purple) and CIV in-gel activity (brown) are shown in Figure 3A. We were able to visualize the minimal supercomplex CI_1_/CIII_2_/CIV_1,_ as well as larger assemblies with increased size and increased densitometric signal for complex IV which we designated as CI_1_/CIII_2_/CIV_2_ and CI_1_/CIII_2_/CIV_3_. 3xTg-AD mice had significant reductions in levels of the minimal mSC CI_1_/CIII_2_/CIV_1_ and increases in mSC containing higher amounts of CI (Figure 3B,D) and CIV (Figure 3C,E) with increases in bands consistent with CI_1_/CIII_2_/CIV_2–3_ supercomplexes. Additional representative gels are provided in Supplemental Figure S1B.

ETC reorganization in 3xTg-AD mice was not associated with alterations in mitochondrial ETC protein expression as assessed by western blot with an antibody cocktail to specific ETC protein subunits (Supplemental Figure S2A–C) or changes in mitochondrial number as assessed by mt-DNA content (Supplemental Figure S2D).

**Figure 4:**
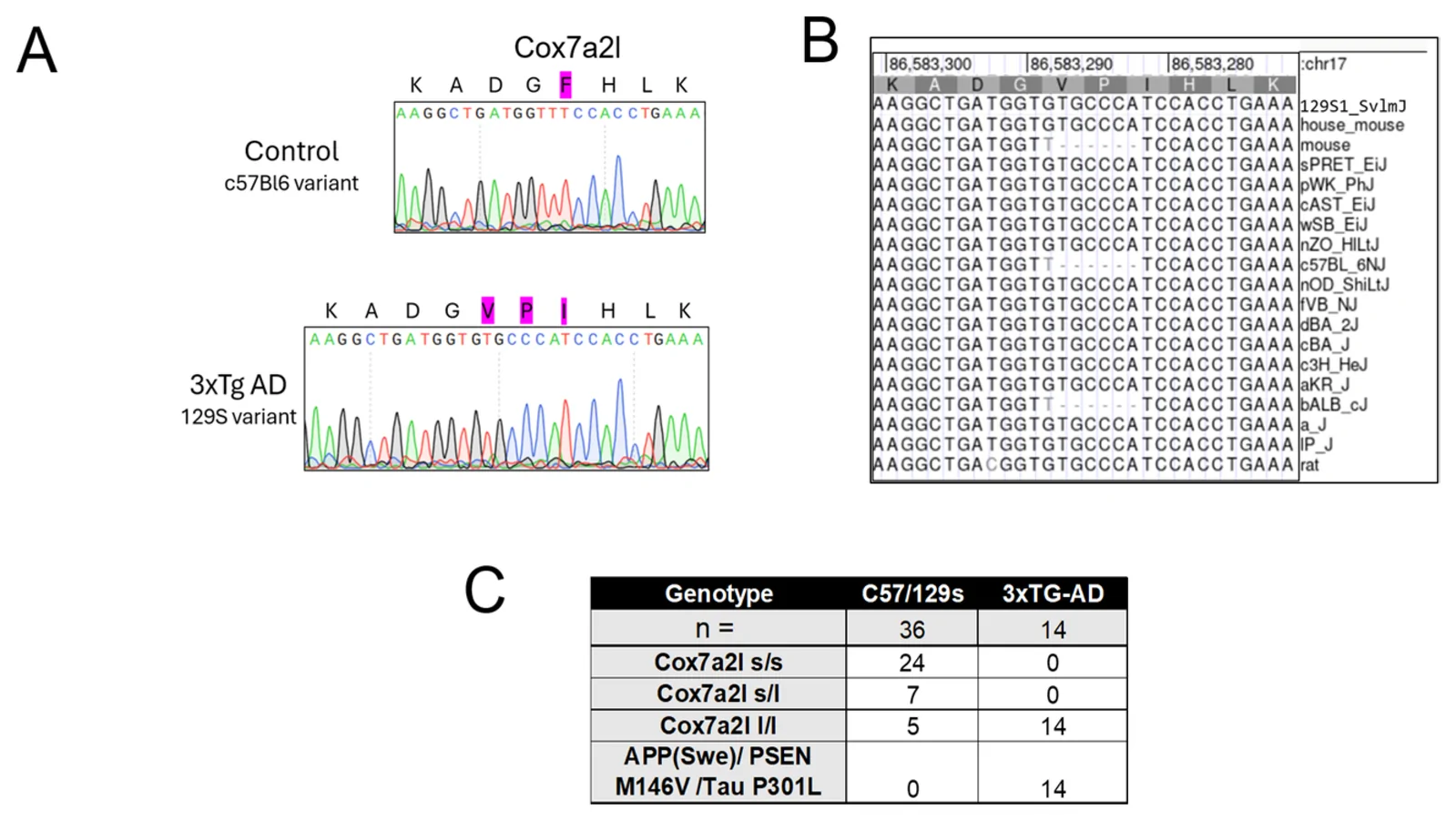
Control and 3xTg AD express different *Cox7a2l* variants. (**A**) Representative sequence data from control and 3xTg mice for *Cox7a2l* around the variable region in 129S and c57BL6 mice. Pink highlights show sequence differences. (**B**) Alignment of *Cox7a2l* mouse strain sequences from UCSC genome browser using the mouse strain assemblies. (**C**) Frequency of short (s) and long (l) variants in control (C57Bl6/129s mice) and 3xTg-AD.

Given such significant changes in the 3xTg-AD mice regarding the additional CIII assembly and larger mSCs, as well as the role of *Cox7a2l* mutation in drastically affecting mSC supercomplex formation and CIII_2_/IV complex formation in c57Bl6 mice [19], we sequenced the *Cox7a2l* gene for a subset of 3xTg-AD and control animals with and without the 650 kD CIII band. We found that control mice carried a *Cox7a2l* variant with a two amino acid deletion (VP) and isoleucine-to-phenylalanine (I to F) mutation compared to the 3xTg-AD mice (Figure 4A). Comparison of these genetic sequences to the mouse strain assemblies in the UCSC genome browser demonstrated that 3xTg-AD mice express the 129S variant of the fully functional *Cox7a2l*, while control lines predominantly express the C57Bl6 variant, which harbors the VP del and I72F mutation (Figure 4B). We then performed a retrospective genotyping analysis to distinguish short and long *Cox7a2l* (Transnetyx, Cordova, TN, USA) on the 12–15-month-old mice described in Figure 1, Figure 2 and Figure 3 (11 Con, 9 3xTg-AD), 17 additional 12–15-month control animals used in an unrelated drug study (see uncropped blots and methods), and 14 (9 control, 5 3xTg-AD) untreated 9-month animals from a pilot study. We found that 3xTg-AD animals were exclusively homozygous for the long isoform of *Cox7a2l*, and 24/36 control animals were homozygous for the short isoform of *Cox7a2l*, while 7/36 carried a long and short allele, and 5 were homozygous for the long allele similar to 3xTg-AD mice) (Figure 4C).

**Figure 5:**
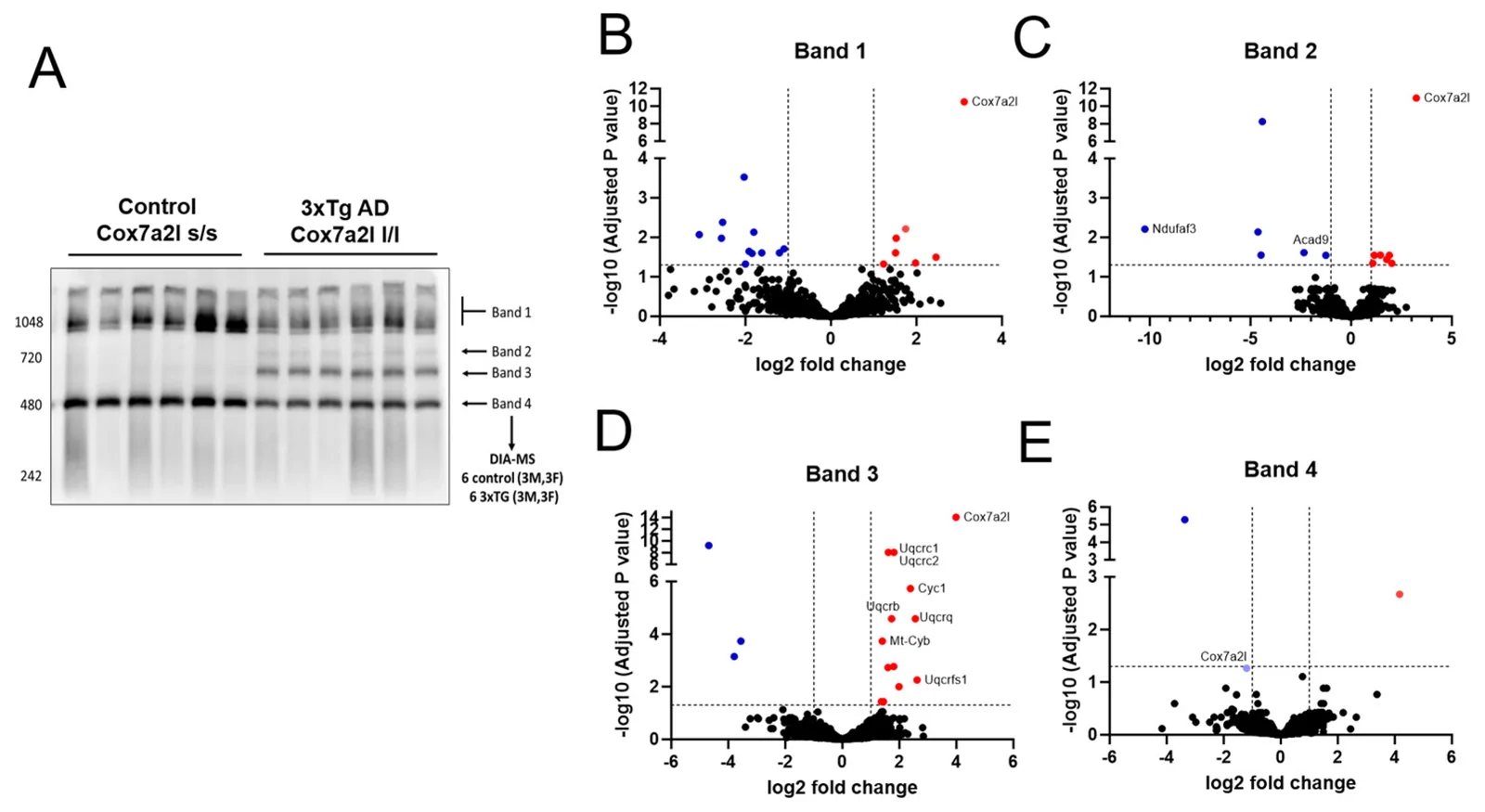
DIA-MS proteomics of control and 3xTg-AD mice reveals COX7a2l is specifically found in the 650 kD complex III band as well as larger mSC. (**A**) BN-PAGE immunoblot of UQCRFS1 (CIII) of 6 control and 6 3xTg-AD (3M,3F) mice. A duplicate gel was used to extract bands from all 12 samples as indicated. (**B**–**E**) Volcano plots of differentially detected proteins in excised bands from 3xTg-AD vs. control mice. (n = 6, lines indicate >1 or <1 fold change and *p* < 0.05 by Benjamini-Hochberg corrected *t*-test. Red = upregulated, blue = down regulated.

To determine the identity of the ~650 kD band unique to 3xTg-AD mice, we excised bands from an unprocessed BN-PAGE gel using a duplicate BN-PAGE immunoblot to determine migration level of relevant Complex III assemblies. Groups included isolated mitochondria from six control (*Cox7a2l* −/−) and six 3xTg-AD (*Cox7a2l* +/+) mice. Bands as indicated in Figure 5A were excised, proteins purified and trypsin digested for DIA-MS. The most changed protein in all the CIII supercomplex bands (bands 1–3) was Cox7a2l the CIII_2_/IV promoting the mSC assembly factor (Figure 5B–D). Differential proteins extracted from the 650 kD CIII band region (band 3) contained mostly increased CIII proteins in addition to Cox7a2l (Figure 5C and Table 1). Band 4, corresponding to the CIII_2_ dimer, had a trend for increased Cox7a2l in control mice (Figure 5D and Table 2, *p* = 0.054). In addition, there was a trend for decreased expression of the alternative mutually exclusive COX7a isoform COX7a2 (which has been shown to promote less tightly bound mSC) in band 3 (*p* = 0.09) and significant decreases in CI assembly factors (ACAD9 and NDUAF3) in band 2 in 3xTg-AD mice compared to control mice (Table 2). Note that comigration in a BN-PAGE gel does not necessarily indicate binding and differentially identified proteins could be associated with different complexes.

As APP was one of the differentially expressed proteins present in excised band 3, we further investigated potential CIII and APP binding in the 3xTg-AD mouse. Probing a duplicate BN-PAGE gel to Figure 5A, with a c-terminal anti-APP antibody (cell signaling), we found that APP comigrated as a distinct band with the 650 kD complex III assembly (Figure S3A) in 3xTg-AD mice. Other native forms of APP in mitochondrial proteins presented as a relatively faint smear from ~150–500 kD in both control and 3xTg-AD mice. We further investigated this potential interaction and found that UQCRFS1, a core component of complex III, coimmunoprecipitated a c-terminal fragment of APP and vice versa (Supplemental Figure S3B). Interestingly, UQCRFS1 pulled down and enriched an apparent ~40 kD and ~15 kD C-terminal fragment of APP, and APP immunoprecipitation pulled down a similar 40 kD band recognized by UQCRFS1 antibody, which may suggest the presence of an APP/UQCRFS1 complex resistant to standard SDS/2-ME denaturation, with a size consistent with bound UQCRFS1 (~25 kD) and the C99 fragment of APP (~15 kD).

**Figure 6:**
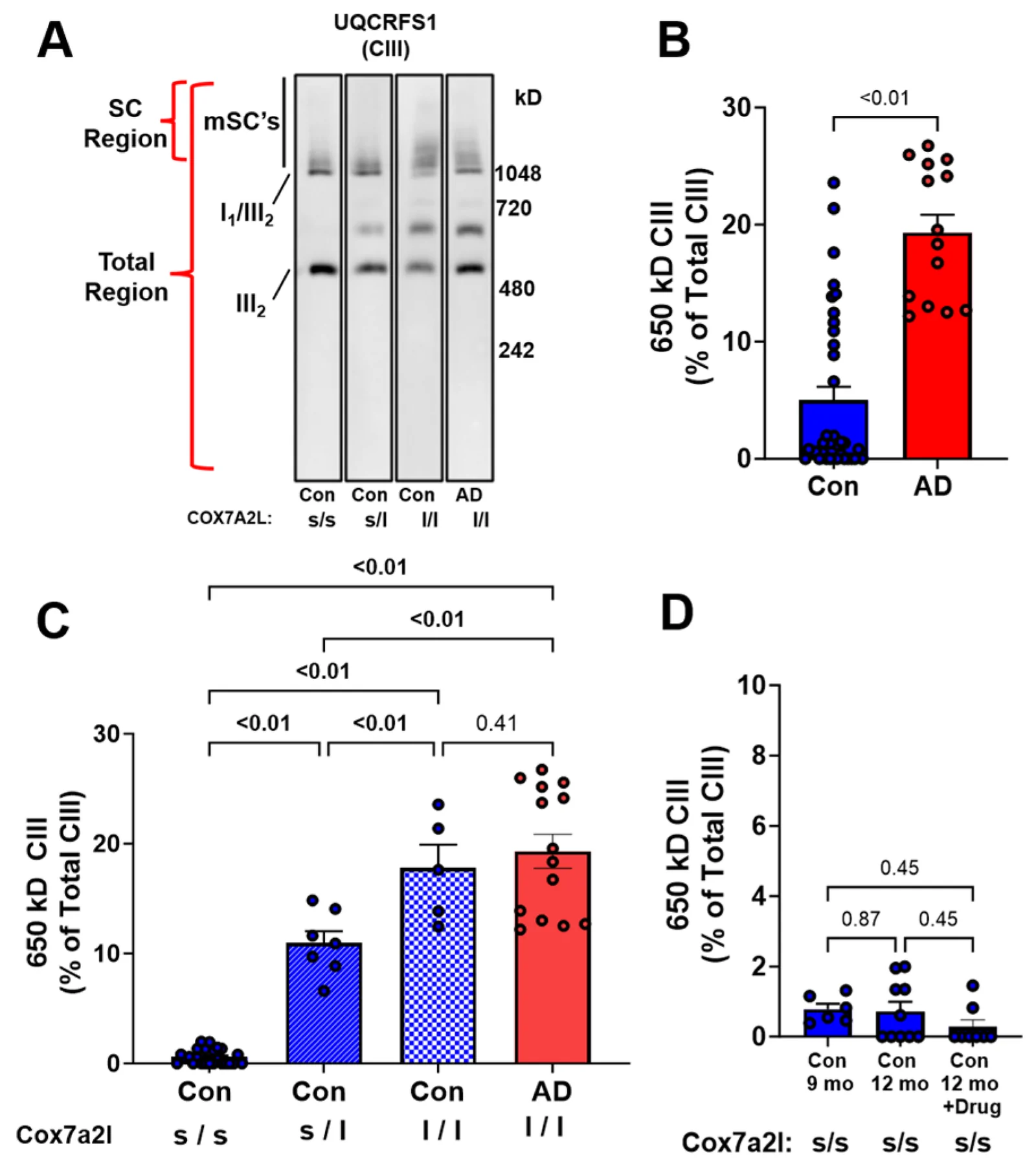
*Cox7a2l* long isoform in control (c57/129S) associates with increased 650 kD CIII assembly. (**A**) Blue Native gel of mitochondria from control mice with 0, 1 or 2 copies of the long (129S) *Cox7a2l* gene. (**B**) Quantitation of 650 kD CIII assembly in control and 3xTg-AD mice regardless of genotype (*p* as indicated, *t*-test). (**C**) Separation of controls into s/s, s/l, and l/l genotypes shows dose-dependent increase in 650 kD CIII assembly similar to 3xTg-AD mice (One Way Anova, Holm Sidak, *p* as indicated, n = 24, 7, 5, 14). (**D**) There were no differences between pooled control groups with *Cox7a2l* s/s genotype (One Way Anova, Holm Sidak, n = 6, 10, 8).

We next performed a retrospective analysis of changes in the complex III 650 kD band based on the *Cox7a2l* genotyping information provided in Figure 4C. All mice were subjected to BN-PAGE and immunoblot for the complex III protein UQCRFS1 as in Figure 2. Control animals were grouped by *cox7a2l* status (s/s: n = 14, s/l: n = 7, l/l: n = 5) and run with AD animals. Control mice that harbored the long *Cox7a2l* allele exhibited increased assembly of the 650 kD CIII band (Figure 6A). When control animals were combined regardless of genotype there was significant variability in 650kD CIII expression (Figure 6B). Separation by genotype showed significant increases in heterozygous animals vs. *Cox7a2l* s/s and further increases in homozygous Cox7a2l l/l control animals. Homozygous *Cox7a2l* l/l mice were not statistically different than 3xTg-AD mice (Figure 6C). There were no differences between the control *Cox7a2l* s/s mice in the 9-month, 12-month, or 12-month + drug treatment group.

**Figure 7:**
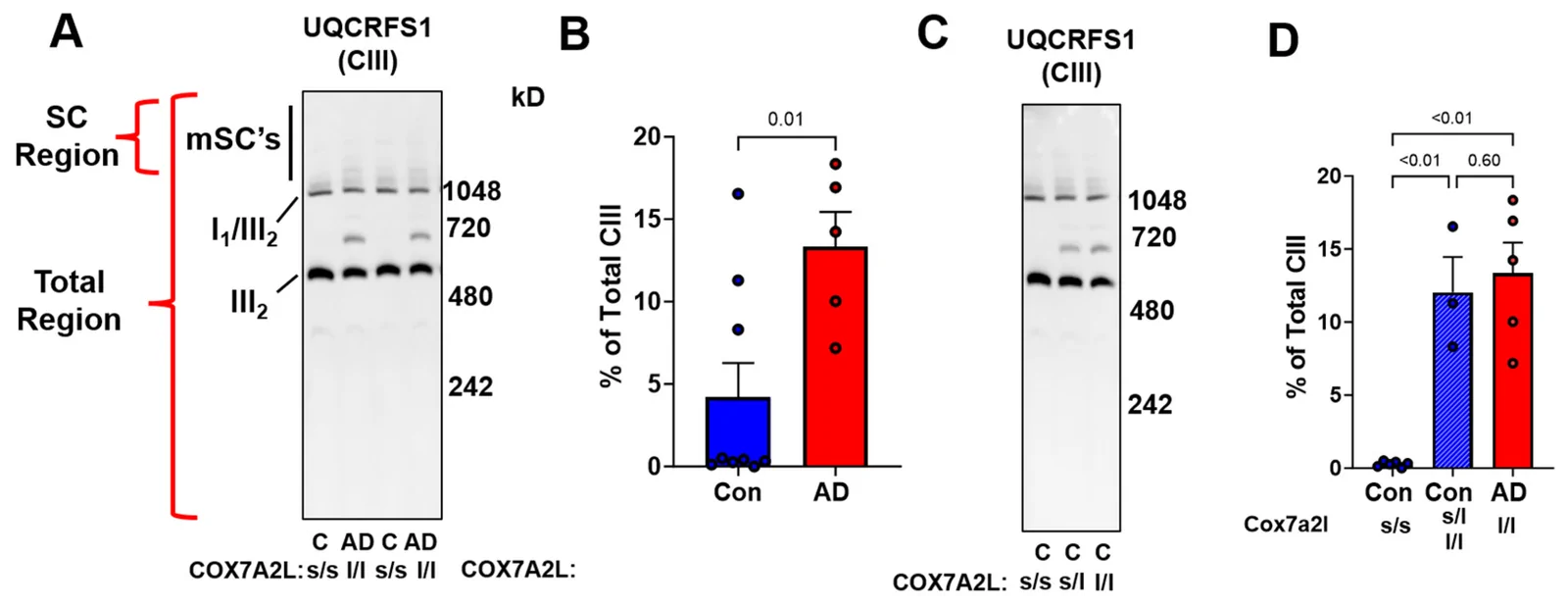
Heart tissue has similar CIII patterns to brain in control and 3xTg-AD mice, and Cox7a2l long isoform increases 650 kD CIII assembly in controls. (**A**) Representative BN-PAGE of heart mitochondria probed for complex III. (**B**) Quantitation of 650 kD CIII assembly in control and 3xTg-AD mice regardless of genotype from (**A**) (n = 9 control, 5 AD, *p* as indicated, *t*-test). (**C**) Blue Native gel of mitochondria from control mice with 0, 1 or 2 copies of the long (129S) *Cox7a2l* gene. (**D**) Controls containing 1 or 2 *Cox7a2l* long alleles show increased 650 kD CIII assembly similar to 3xTg-AD mice (One Way Anova, Holm Sidak, *p* as indicated, n = 6, 3, 5).

We further investigated the role of mutant APP and MAPT to contribute to the altered Complex III assembly associated with the *Cox7a2l* l/l genotype, we performed a similar analysis on control and 3xTg-AD heart tissue in the 9-month-old cohort described in Figure 6. In the 3xTg-AD mouse model, mutant APP and MAPT are driven by the Thy1.2 promoter, which does not show expression in heart tissue. Isolated heart mitochondria from control and 3xTg-AD mice showed a similar BN-PAGE complex III pattern (Figure 7A). Combining all samples regardless of *Cox7a2l* genotype showed similar changes to brain mitochondria with large increases in 3xTg-AD mice (Figure 7B). Separation of control animals by *Cox7a2l* genotype status demonstrated increases in the 650 kD CIII band associated with increased copies of the *Cox7a2l* long allele (Figure 7C). Only three control mice carried the long *Cox7a2l* allele (1 s/l, 2 l/l), and they were pooled for comparison analysis, which showed a significant increase in the 650 kD CIII band associated with the *Cox7a2l* long allele that was indistinguishable from 3xTg-AD mice (Figure 7D). Thus, complex III alterations in control vs. 3xTg-AD mixed background mice are likely independent of APP and MAPT mutations. However, the heart does express the knock-in PSEN1 M146V mutation.

**Figure 8:**
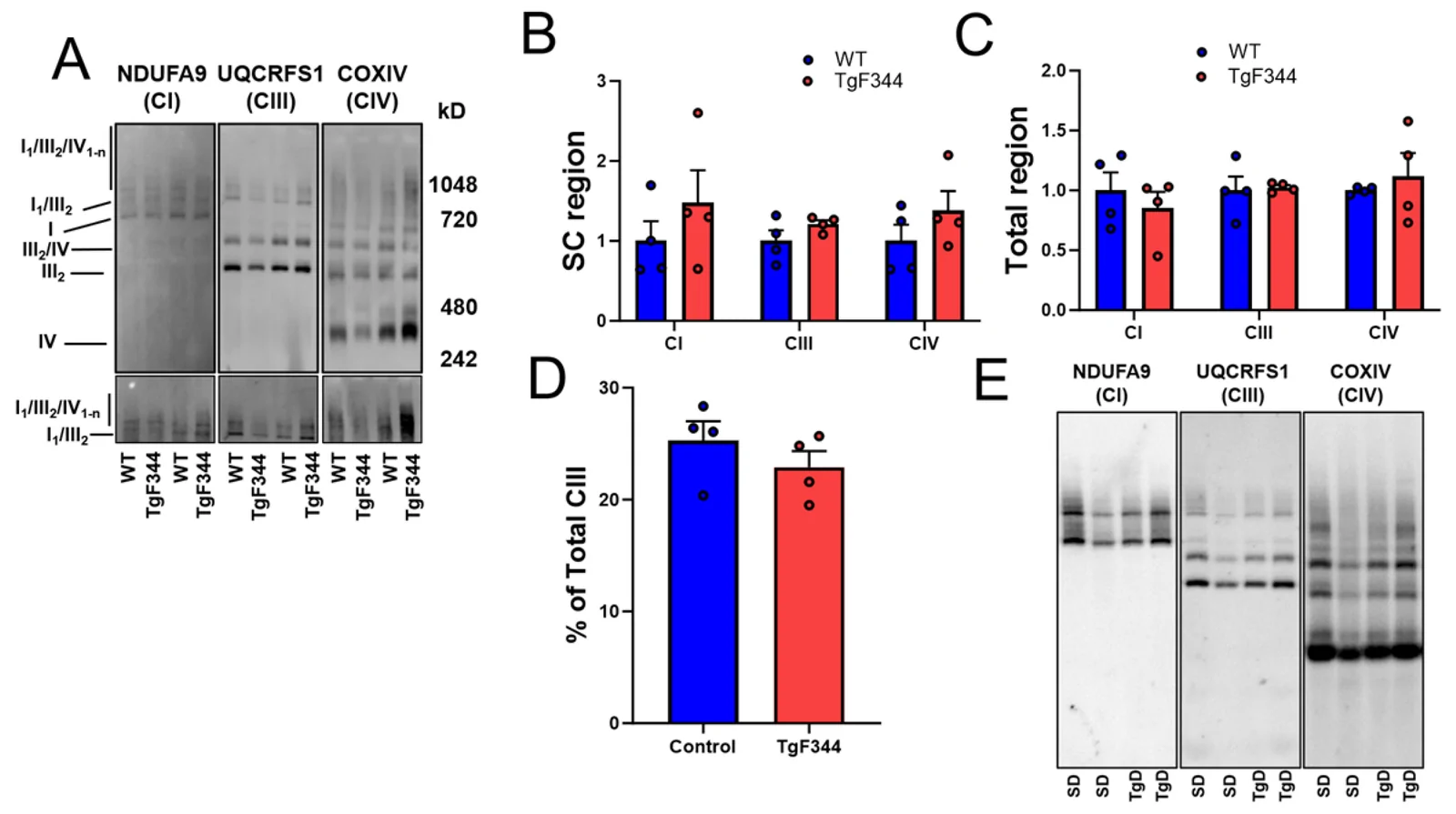
Transgenic rat AD models do not mimic 3xTg-AD mSC and CIII derangements. (**A**) Blue Native gel of control and TgF344 rat (human APP-695 (Swe) and PSEN1) shows the presence of the ~650 kD CIII band in both control and TgF344 rats with no changes in mSC distribution. (**B**–**D**) Quantitation of results in (**A**) (n = 4). (**E**) Similar results to TgF344-SD rats were found in rTgD rat (APP 770-Swe/Dutch) (n = 2).

Finally, as rats only contain the longer *Cox7a2l* variant consistent with 129S mice, we screened additional rat models that over-express APP and produce ADRD pathologies for changes in mSC distribution and the presence/absence of the ~650 kD CIII_2_ band. TgF344 rats with overexpression of human APP (Swedish (KM670/671NL)) and human PSEN1ΔE9 bred to the Sprague Dawley background [24] had robust assembly of the 650 kD CIII band (likely CIII_2_/CIV) and no apparent shifts in larger mSC in the AD rat models vs. control as shown by BN-PAGE (Figure 8A). Quantitation showed no apparent changes in TgF344 rat complex III or mSC assemblies compared to control rats (Figure 8B,C) or any changes in the 650 kD CIII band (Figure 8D). We further investigated an additional ADRD rat model, rTgD (transgenic human APP770 with Swedish and Dutch mutation) on the Sprague Dawley background [25]. BN-PAGE showed no apparent changes in CIII organization or shifts to larger supercomplexes (Figure 8E). These results further support that the drastic changes observed in mSC and CIII_2_/IV assembly in the 3xTg-AD mouse lines are dependent on *Cox7a2l* strain differences and not pathophysiologic differences due to APP or PSEN1 modulation. However, we should note the high variability observed with complex I and IV inclusions in mSC and that this rat study was underpowered to detect more subtle differences in mSC assembly and distribution than those observed in 3xTg-AD mice. This analysis does not rule out that APP or PSEN-related gene expression changes may impact mSC assembly. This data does suggest that the presence of the 650 kD CIII band and large changes in mSC abundance are independent of APP/PSEN modifications in these rat models. These rats do not express mutant MAPT, which may be involved in 3xTg-AD mice supercomplex alterations. However, the rat studies together with the 3xTg-AD heart data and sufficiency of *Cox7a2l* long allele to drive the CIII phenotype collectively strongly support that CIII alterations in 3xTg-AD mice are independent of APP, PSEN1 and MAPT.

## 3. Discussion

In this study, we demonstrate that the widely used 3xTg-AD mouse line maintained on a mixed C57BL/6J:129/SvlmJ (B6;129S) background exhibits pronounced differences in mitochondrial electron transport chain (ETC) supramolecular organization compared with the commonly employed B6129SF2/J control strain. Importantly, our data suggests these differences are influenced by background-strain variation in the mitochondrial supercomplex (mSC) assembly factor *Cox7a2l* rather than by 3xTg-associated expression of APP, PSEN1, or Tau. These findings identify the *Cox7a2l* genotype as a major determinant of ETC organization in this model system and provide important context for interpretation of mitochondrial phenotypes reported in 3xTg-AD mice.

*Cox7a2l* is a well-established regulator of CIII_2_/CIV binding and assembly [16,26]. Because CIII_2_ readily associates with CI and most CI is bound to CIII_2_, Cox7a2l promotes CIV incorporation into both CIII_2_-containing and CI_1_/CIII_2_-containing structures, thereby increasing formation of CIII_2_/CIV_1_ and CI_1_/CIII_2_/CIV_n_ supercomplexes. Modulation of *Cox7a2l* is also associated with substantial physiological effects. *Cox7a2l* knockout mice exhibit reduced exercise capacity, lower VO_2_max, and reduced mitochondrial respiration compared with wild-type animals [27]. Conversely, expression of functional Cox7a2l in C57BL/6 mice increases exercise capacity, VO_2_max, and mitochondrial respiration relative to C57BL/6 mice carrying the nonfunctional variant [28]. In humans, a 3′UTR insertion that increases *Cox7a2l* expression is positively correlated with increased mitochondrial supercomplex abundance, mitochondrial respiration, and cardiorespiratory fitness [28]. Together, these studies establish functional *Cox7a2l* as an important modulator of mSC assembly and bioenergetic performance, including endpoints central to studies of AD-related neuronal pathology and exercise responsiveness. Interestingly, we observed a trend toward improved rotarod performance in 3xTg mice, consistent with previous studies [23], raising the possibility that the long *Cox7a2l* variant may contribute to improved rotarod performance observed in this strain.

Differences among *Cox7a2l* isoforms in specific mouse strains have been previously described [19,29,30,31]. C57BL/6 strains are homozygous for a short 111-amino acid variant containing a two-amino acid deletion and an I72F substitution. BALB mice are the only other known strain reported to carry this variant [19,32]. In contrast, all other available sequenced mouse strains (Figure 6B) carry the full-length 113-amino acid variant, which corresponds to the *Cox7a2l* sequence present in humans and almost all other mammals. The shorter C57BL/6 variant has been shown to impair CIII_2_/CIV_1_ assembly and markedly reduce CI-dependent mitochondrial respiration in C57BL/6 liver tissue [19]. These prior observations are consistent with our finding that the *Cox7a2l* genotype strongly shapes ETC supramolecular architecture in the mixed background 3xTg model system.

B6;129S 3xTg mice (JAX 034830) exhibited a supercomplex profile distinct from that of their commonly used B6129SF2/J control strain (JAX 101045), including greater CIII/CIV interaction and a shift toward larger mSC assemblies with increased CIV incorporation. Notably, B6129SF2/J control mice readily form respiratory supercomplexes, with increased CI_1_/CIII_2_/CIV_1_ assemblies compared to 3xTg mice but display considerably fewer larger CI_1_/CIII_2_/CIV_2–3_ supercomplexes. These results are consistent with previous supercomplex assembly studies in HEK cell lines with and without *Cox7a2l* knockout [26]. Recent in situ cryo-EM studies by Zheng et al. showed that multiple mSC structures are readily detected in pig heart mitochondria, with CI_1_/CIII_2_/CIV_1_ comprising more than 60% of the mSC population, CI_1_/CIII_2_/CIV_2_ approximately 20%, and CI_2_/CIII_2_/CIV_2_ approximately 8%; based on these structural data, the authors hypothesized that the additional CIV within CI_1_/CIII_2_/CIV_2_ could interfere with electron transfer between CI and CIII [11]. This may be relevant to the 3xTg mice examined here, which showed significantly more CI_1_/CIII_2_/CIV_2–3_ mSCs. However, the functional impact of these higher-order CIV-containing assemblies remains to be experimentally defined.

We also provide evidence that a C-terminal fragment of APP associates with the 650 kD complex III-containing structure in 3xTg-AD mice (Supplemental Figure S3). This observation supports the compelling hypothesis that APP overexpression, altered processing, or aberrant mitochondrial trafficking may influence mitochondrial ETC assembly or organization. Given the strong effect of *Cox7a2l* genotype identified in the present study, future experiments using appropriately matched genetic backgrounds will be required to determine if APP-complex III binding has biological or pathophysiological significance in AD-related mitochondrial dysfunction.

There is significant broader impact of *Cox7a2l* variation in these specific mouse lines as 3xTg mice are among the most common and well-characterized preclinical AD mouse models. First published in 2003, at the time of writing there are 1724 papers listed in PubMed Central (search: 3xTg and variations (3x-Tg, 3x Tg)) and 212 papers focused on mitochondrial changes (search: 3xTg AND mitochondria) [20]. Jackson Laboratory maintains the 3xTg mutations on three backgrounds: B6;129S mixed background (JAX 034830), C57BL/6J background (JAX 033930), and 129S4 background (JAX 031988). Through literature survey, the 3xTg B6;129S strain is the most frequently used by far. Our finding that ETC organization is strongly influenced by strain-dependent *Cox7a2l* allele variation highlights the need for careful interpretation of mitochondrial phenotypes in studies employing mixed-background 3xTg mice and B6129SF2/J controls. *Cox7a2l*-dependent modification of mSC assembly has been shown to affect ROS, mitochondrial Ca^2+^ handling, and respiration [17,33]. In addition, mSC organization can modify mitochondrial inner membrane curvature and potentially cristae architecture [11]. Thus, the B6;129S 3xTg model presents a substantial genetic confounder for studies seeking to attribute alterations in mSC assembly, oxidative stress, respiration, or metabolism specifically to AD-like pathology. These data also suggest that caution is warranted when using C57BL/6J-background mice for studies focused on physiological regulation of mSC assembly, because the C57BL/6J *Cox7a2l* variant differs from the allele present in humans and most other mammals. To minimize these concerns, we recommend use of the available 3xTg line on the 129S4 background (JAX 031988), which carries the longer *Cox7a2l* variant, for future studies of AD-related mitochondrial structure and function.

The results presented in this manuscript neither support nor exclude the possibility that mitochondrial supercomplexes may be altered in Alzheimer’s disease. Furthermore, this study does not disprove that mutant APP, PSEN1 or Tau may alter mitochondrial supercomplexes and subsequent mitochondrial function in 3xTg-AD mixed background mice. This study solely provides compelling evidence that the most commonly used control strain (C57Bl6/129S) are inappropriate for mixed background 3xTg-AD mice in studies investigating ETC organization and mSC-relevant mitochondrial function. However, despite the ETC organization differences that correlated well with *Cox7a2l* genotype, it is possible that gene expression changes in 3xTg mice due to mutant APP, PSEN, MAPT, related downstream expression changes, or protein alterations such as those detected in the DIA-MS analysis may play a role. Definitive conclusions differentiating *Cox7a2l* variation from 3xTg-related gene expression will require comparative studies between control and 3xTg-AD mice on both the short (C57) and long (129S) *Cox7a2l* background.

### Limitations

Our colony was established from two breeding pairs each of 3xTg and control mice. We observed no instance of mSC assembly consistent with the short *Cox7a2l* variant in more than 21 3xTg animals, including animals from studies not described here. However, it remains possible that individual mice from the Jackson Laboratory 3xTg colony may carry this variant. In contrast, 12 of 36 control animals from multiple studies exhibited CIII assemblies consistent with the long 129S variant, indicating limited retention of this allele in the F2-generation control colony. Because allele segregation is expected within an F2-derived population, this variability should be accounted for in evaluating results of mitochondrial studies using these strains.

## 4. Materials and Methods

### 4.1. Animals

All studies were approved by the University of Rhode Island Institutional Animal Care and Use Committee (IACUC). Mice were from Jackson laboratories, and 3xTg-AD (034830-JAX) and B6129SF2 mice (101045-JAX) were used as controls. Colonies were initiated with 2 pairs each of 3xTg-AD and control strains. Lines were maintained independently. Mice were aged to 12–15 months for all experiments, and male and female mice were used. Prior to behavioral testing and sacrifice, both control and 3xTg mice were I.P. injected with ~100 ul 10% DMSO in sterile PBS every other day for 2 weeks (mice were vehicle controls for an unrelated study). Additional cohorts were used for some analyses (Figure 6 and Figure 7) including nine-month-old 3xTg-AD and control mice as well as controls from the 12–15 month drug treatment study (Figure 6). All mice from these cohorts were from the same founder animals as the 12–15 mo control and 3xTg-AD vehicle controls. TgF344 rats (n = 4) and wt controls (n = 4) were 18 months old and obtained from Rat Resource and Research Center (Strain: F344-Tg (Prp-APP, Prp-PS1)19/Rrrc, Columbia, MO, USA) and backcrossed for ten generations onto the Sprague Dawley background. These rats overexpress human APP-695 with Swedish mutation and human PSEN1 with exon 9 deletion [24]. rTg-D rats (n = 2, 13 months old) which overexpress Human APP-770 with Swedish and Dutch mutation and are previously described [25]. rTgD rats were 13 months old. All rat lines were on a Sprague Dawley background.

### 4.2. Open Field Maze

Mice were individually placed inside an opaque cylinder positioned in the center of the open field arena. Each 300-s trial automatically initiates upon the removal of the cylinder. Animal locomotive activity and tracking movements were monitored via an automated infrared photobeam array and recorded using Fusion v6.5 software (Omnitech Electronics, Inc., Columbus, OH, USA). On Day 1, animals underwent an initial trial to allow for environmental acclimation. Experimental data collection was conducted during a subsequent assessment on Day 2.

### 4.3. Rotarod

Mice were evaluated for motor coordination and balance using an automated rotarod system. Testing was conducted over two days, consisting of three trials per day. The rotarod was operated in an accelerating mode, initiating at an initial speed of 4 RPM and increasing by 0.12 RPM every 1 s. For each animal, the peak latency to fall across the three experimental trials on Day 2 was utilized for definitive statistical analysis.

### 4.4. Barnes Maze

Spatial learning and memory were evaluated using a 20-hole mouse Barnes maze apparatus (Stoelting Co., Wood Dale, IL, USA). The maze configuration consisted of a single functional escape hole paired with 19 identical non-functional (false) holes. Animal movement and head-entry tracking were captured and analyzed using ANY-maze behavioral tracking software (Stoelting Co., version 7.67). Mice underwent an acquisition phase consisting of two 5-min (300-s) trials per day across 5 consecutive days. The primary metric reported is the primary latency (the time elapsed before the first head entry into the escape hole), with the lowest latency achieved between the two daily trials reported as the definitive value for each day.

### 4.5. Mitochondrial Isolation

Blue native and clear native-polyacrylamide gel electrophoresis BN or CN-PAGE was performed similarly to our previous publications [34,35]. In mice, ~50 mg of frontal lobe, stored in −80 °C were finely minced, and transferred to 1 mL of ice-cold mitochondrial isolation buffer (MIB; 0.28 M sucrose, 10 mM HEPES, and 2 mM EDTA, pH 7.4) containing protease and phosphatase inhibitor cocktails (Sigma-Aldrich). Tissue homogenization was performed using a 2 mL Dounce homogenizer with 15 passes of both the loose and tight-fitting pestles. Mitochondria were enriched by differential centrifugation. Homogenates were first centrifuged at 2000× *g* for 5 min at 4 °C to remove nuclei and cellular debris. The resulting supernatant was subsequently centrifuged at 13,000× *g* for 10 min at 4 °C to pellet mitochondria. Mitochondrial pellets were washed twice with fresh MIB. The final pellet was resuspended in 100 μL of MIB, and protein concentration was determined using a bicinchoninic acid (BCA) assay (Pierce, Thermo Fisher Scientific, Waltham, MA, USA).

### 4.6. Blue and Clear Native Polyacrylamide Gel Electrophoresis (BN/CN-PAGE)

Mitochondria were solubilized in MIB containing 1% digitonin at a volume of 2 μL per μg mitochondrial protein. Following solubilization, protein concentrations were measured by BCA assay. Equal amounts of mitochondrial protein were mixed with NativePAGE sample buffer (Invitrogen, Carlsbad, CA, USA) supplemented with 1% digitonin and 0.25% Coomassie G-250. Approximately 10 μg of mitochondrial protein per lane was loaded onto 4–16% NativePAGE gradient gels (Invitrogen). Electrophoresis was performed initially at 150 V for 60 min using NativePAGE anode buffer and dark blue cathode buffer, followed by separation at 250 V for 180 min after replacement with light blue cathode buffer according to the manufacturer’s recommendations. Proteins were transferred to nitrocellulose membranes and immunoblotted using antibodies against NDUFA9 (Complex I; 1:1000, Abcam: 14713, Waltham, MA, USA), UQCRFS1 (Complex III; 1:1000, Abcam: 14746), and COXIV (Complex IV; 1:1000, Abcam: 14744). CN-PAGE was carried out using the same sample preparation procedure as BN-PAGE with modifications described previously. Electrophoresis was conducted at 150 V for 60 min using light blue cathode buffer, followed by 250 V for 180 min using clear cathode/running buffer. Upon completion of electrophoresis, gels were briefly rinsed in ice-cold distilled water and subjected to in-gel enzymatic activity staining. For Complex IV activity, gels were incubated in phosphate buffer (38 mM Na_2_HPO_4_ and 12.3 mM NaH_2_PO_4_, pH 7.4) containing 0.5 mg/mL 3,3′-diaminobenzidine and 1 mg/mL cytochrome c. Complex I activity was assessed using a staining solution consisting of 0.1 mg/mL NADH and 2.5 mg/mL nitro blue tetrazolium chloride prepared in 2 mM Tris buffer (pH 7.4). Gels were incubated in 20 mL of the appropriate reaction mixture until visible color development was achieved (typically 30–60 min). Reactions were terminated by immersion in 10% acetic acid, followed by extensive washing with distilled water prior to imaging and analysis.

For rat BN-PAGE studies, brains were pulverized with a mortar and pestle under liquid nitrogen. 10 mg of pulverized tissue were solubilized in 50 μL of MIB buffer with 1% digitonin for 1 h. Insoluble debris was removed via centrifugation at 20,000× *g*. Soluble protein concentration was assessed via BCA and 20 μg were loaded per well.

For both BN and CN-PAGE, mSC densitometry values are expressed as % in supercomplexes, which is the densitometry values of the indicated mSC or entire mSC region divided by the total signal for the respective complex multiplied by 100.

### 4.7. MS/MS Analysis of Gel Fragments

Duplicate BN-PAGE gels were run and one immunoblotted for UQCRFS1 to visualize complex III and used as a guide for excision of specified bands. Gel digestion and DIA-MS were performed by the IDEA National Resource for Proteomics at University of Arkansas Medical Center. Each BN-PAGE gel band was subjected to in-gel trypsin digestion as follows. Gel segments were destained in 50% methanol (Thermo Fisher Scientific, Waltham, MA, USA), 50 mM ammonium bicarbonate (Sigma-Aldrich, St. Louis, MO, USA), followed by reduction in 10 mM Tris [2-carboxyethyl]phosphine (Pierce) and alkylation in 50 mM iodoacetamide (Sigma-Aldrich). Gel slices were then dehydrated in acetonitrile (Fisher), followed by the addition of 100 ng porcine sequencing grade modified trypsin (Promega, Madision, WI, USA) in 50 mM ammonium bicarbonate (Sigma-Aldrich) and incubation at 37 °C for 12–16 h. Peptide products were then acidified in 0.1% formic acid (Pierce). Tryptic peptides were then separated by reverse phase XSelect CSH C18 2.5 um resin (Waters, Mildord, MA, USA) on an in-line 150 × 0.075 mm column using an UltiMate 3000 RSLCnano system (Thermo Fisher Scientific). Peptides were eluted using a 60 min gradient from 98:2 to 65:35 buffer A:B ratio. Eluted peptides were ionized by electrospray (2.2 kV) followed by mass spectrometric analysis on an Orbitrap Eclipse Tribrid mass spectrometer (Thermo Fisher Scientific). MS data were acquired using the FTMS analyzer in profile mode at a resolution of 120,000 over a range of 375 to 1400 m/z with advanced peak determination. Following HCD activation, MS/MS data were acquired using the ion trap analyzer in centroid mode and normal mass range with a normalized collision energy of 30%. Proteins were identified by database search using MaxQuant (Max Planck Institute, Munich, Germany, version v2.6.4.0) label-free quantification with a parent ion tolerance of 2.5 ppm and a fragment ion tolerance of 0.5 Da. Scaffold Q+S (Proteome Software, version 5.3.4) was used to verify MS/MS based peptide and protein identifications. Protein identifications were accepted if they could be established with less than 1.0% false discovery and contained at least 2 identified peptides. Protein probabilities were assigned by the Protein Prophet algorithm [36].

### 4.8. mtDNA

DNA was isolated with a Qiagen DNAeasy kit according to manufacturer instructions. Mitochondrial DNA (mtDNA) copy number was subsequently assessed via quantitative PCR (qPCR) targeting the mitochondrial gene mt-Co1 (cytochrome c oxidase subunit I). Mice DNA from the brain was isolated with a Qiagen DNAeasy kit according to manufacturer instructions. (Forward 5′-TGC TAG CCG CAG GCA TTA C-3′; Reverse 5′-GGG TGC CCA AAG AAT CAG AAC-3′), and nuclear DNA with NDUFV1 (Forward 5′-CTT CCC CAC TGG CCT CAA G-3′; Reverse 5′-CCA AAA CCC AGT GAT CCA GC-3′).

### 4.9. SDS-PAGE

Mitochondrial ETC protein content was visualized using SDS-PAGE and immunoblot with an OXPHOS antibody cocktail (1:1000; Abcam: ab110413), NDUFS1 (1:1000; Cell Signaling Technology: 70264, Danvers, MA, USA) and HSP60 (1:1000; Cell Signaling Technology: 12165).

### 4.10. Statistics

Differences between 3xTg-AD and control mice were determined using unpaired *t*-test vs. control for behavioral and mSC assessment. DIA-MS proteomics analysis between groups used Benjamini–Hochberg corrected *t*-tests. *p* < 0.05 was considered significant.

## Figures and Tables

**Table 1:** Increased proteins in 3xTg-AD in excised bands vs. control.

Increased in 3xTg vs. Control											
Band 1			Band 2			Band 3			Band 4		
Protein	Log2 FC	B-H Adj P	Protein	Log2 FC	B-H Adj P	Protein	Log2 FC	B-H Adj P	Protein	Log2 FC	B-H Adj P
Cox7a2l	3.12	3.2 × 10^−11^	Cox7a2l	3.24	1.2 × 10^−1^	Cox7a2l	3.99	8.8 × 10^−15^	Stoml2	4.17	2.1 × 10^−3^
Stk39	2.46	3.2 × 10^−2^	Dnm3	1.46	2.8 × 10^−2^	Uqcrc2	1.81	8.8 × 10^−9^			
Iars1	1.98	4.5 × 10^−2^	Dync1i2	1.89	2.9 × 10^−2^	Uqcrc1	1.62	8.8 × 10^−9^			
Aldoc	1.75	6.2 ×10^−3^	Pacsin1	1.16	2.9 × 10^−2^	Cyc1	2.39	1.9 × 10^−6^			
Epb41l1	1.53	1.1 × 10^−2^	Cfl1	1.77	3.6 × 10^−2^	Uqcrq	2.56	2.6 × 10^−5^			
Hexb	1.51	2.5 × 10^−2^	Zw10	2.02	4.5 × 10^−2^	Uqcrb	1.73	2.6 × 10^−5^			
Sfxn1	1.23	4.8 × 10^−2^	Kif1a	1.08	4.5 × 10^−2^	Mt−Cyb	1.41	1.9 × 10^−4^			
						App	1.81	1.7 × 10^−3^			
						Tfg	1.6	1.9 × 10^−3^			
						Uqcrfs1	2.62	5.6 × 10^−3^			
						Pclo	1.99	9.9 × 10^−3^			
						Hcfc1	1.44	3.7 × 10^−2^			
						Ap1g1	1.37	3.7 × 10^−2^			

Green highlight Cox7a2l, blue highlight CIII proteins.

**Table 2:** Decreased proteins in 3xTg-AD in excised bands vs. control.

Decreased in 3xTg vs. Control											
Band 1			Band 2			Band 3			Band 4		
Protein	Log2 FC	B-H Adj P	Protein	Log2 FC	B-H Adj P	Protein	Log2 FC	B-H Adj P	Protein	Log2 FC	B-H Adj P
Slc17a7	−2.03	3.0 × 10^−4^	Sirpa	−4.41	5.6 × 10^−9^	Mpc1	−3.56	1.9 × 10^−4^	Sirpa	−3.36	5.3 × 10^−6^
Mthfd1l	−2.53	4.2 × 10^−3^	Ndufaf3	−10.24	6.1 × 10^−3^	Sirpa	−4.68	6.0 × 10^−10^	Cox7a2l	−1.19	5.4 × 10^−2^
Ppib	−1.80	7.4 × 10^−3^	Mdh1	−4.63	7.3 × 10^−3^	Trappc9	−3.79	7.1 × 10^−4^			
Cdk5	−3.08	8.5 × 10^−3^	Acad9	−2.35	2.4 × 10^−2^	Cox7a2l	−0.86	9.0 × 10^−2^			
Glg1	−2.56	1.1 × 10^−2^	Vat1l	−4.47	2.8 × 10^−2^						
Septin8	−1.09	2.0 × 10^−2^	Rab5c	−1.26	2.9 × 10^−2^						
Napg	−1.91	2.3 × 10^−2^									
Slc32a1	−1.20	2.5 × 10^−2^									
Rps4x	−1.62	2.5 × 10^−2^									
Efr3b	−1.84	2.6 × 10^−2^									
Letm1	−2.00	4.8 × 10^−2^									

Green highlight Cox7a2l or Cox7a2, orange highlight CI assembly proteins.

## Data Availability

All DIA-MS data are included as spreadsheets in the Supplemental Materials. Note, in the Supplemental Materials, animals are also listed with “drug”. The “drug”-treated animal data will likely be incorporated into future publication and thus identity of substances administered is withheld. All other data available upon request.

## Supplementary Materials

The following supporting information can be downloaded at https://www.mdpi.com/article/10.3390/ijms27177710/s1.

## Abbreviations

The following abbreviations are used in this manuscript:

Aβ	Amyloid Beta
AD	Alzheimer’s Disease
APP	Amyloid Precursor Protein
BN-PAGE	Blue-Native Polyacrylamide Gel Electrophoresis
CI	Complex I
CIII	Complex III
CIV	Complex IV
CN-PAGE	Clear-Native Polyacrylamide Gel Electrophoresis
DIA-MS	Data independent acquisition mass spectrometry
ETC	Electron Transport Chain
mSC	Mitochondrial supercomplexes
PSEN1	Presenilin 1
ROS	Reactive Oxygen Species
