# Supplementary material for: Cox7a2l Variation Influences Complex III and Mitochondrial Supercomplex Differences in 3xTG-AD Mixed-Background Mice
Source: Int J Mol Sci. 2026 Aug 28;27(17):7710. doi: 10.3390/ijms27177710 (PMC13566326; doi:10.3390/ijms27177710)
Supplement: Supplementary file 1 [file ijms-27-07710-s001.zip › Supplemental Figures.pdf]

Figure S1

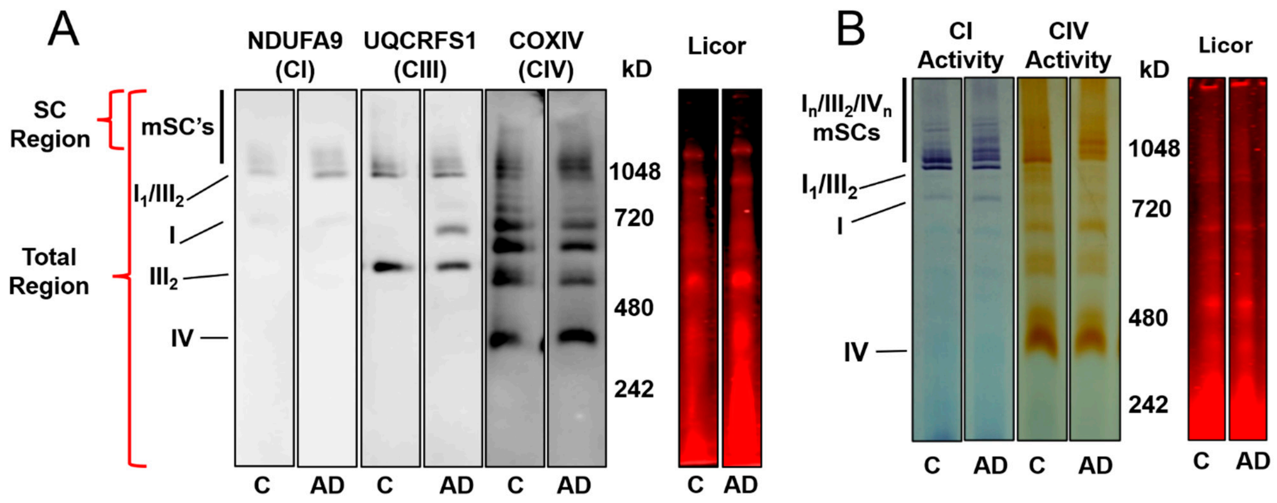

Figure S1: A) Additional representative blots of BN-PAGE of mouse brain mitochondria and far red-imaging of Coomassie stained proteins in gel (Licor). **B**) Additional representative gels of in-gel activity assays for CI and CIV, showinga greater abundance of larger mSCs in 3xTg-AD mice vs control mice, and protein load control (licor).

Figure S2

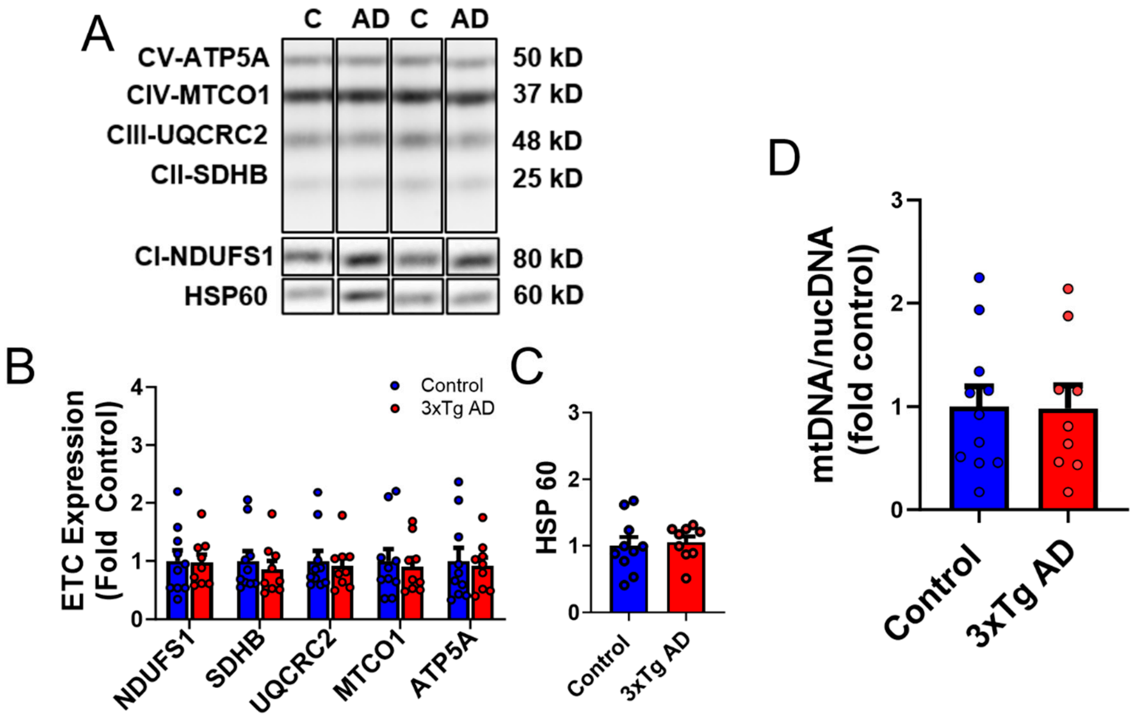

Figure S2: Control and 3xTg-AD mice have similar ETC protein expression and mitochondrial content. A) Assessment of representative ETC-subunits in control and 3xTg-AD mice using OXPHOS antibody cocktail and SDS-PAGE. **B,C**) Quantitation of data in A (n=10,9, * P<.05 T-test +/- SEM). **D**) Mitochondrial content in control and 3xTg-AD mice (n=11,9, * P<.05 T-test +/- SEM).

Figure S3

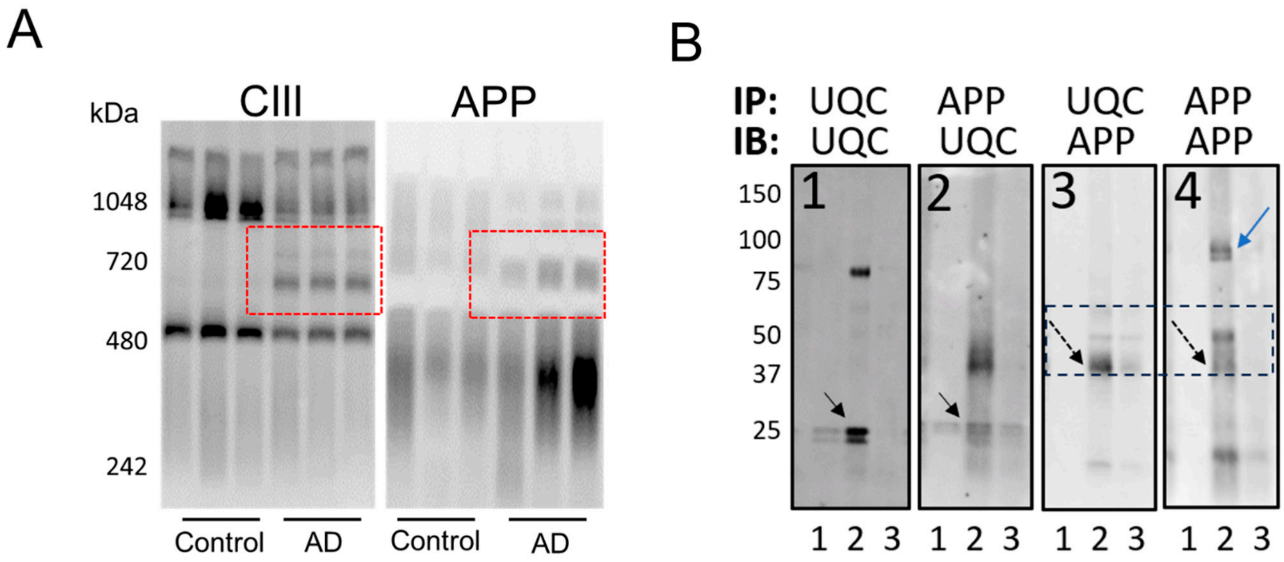

Figure S3: 3xTg-AD mice show comigration of APP with 650 kD CIII band. A) Duplicate BN-PAGE gels of isolated brain mitochondria stained for UQCRFS1 and APP (note UQCRFS1 image is same as Figure 4) show a distinct APP band that comigrates with UQCRFS1 (n=6). **B**) Reciprocal immunoprecipitation and immunoblot of complex III component UQCRFS1 (UQC) or APP demonstrates binding of UQCRFS1 and a c-terminal fragment of APP of 45 kD or a UQC/APP.
